# Research on eight machine learning algorithms applicability on different characteristics data sets in medical classification tasks

**DOI:** 10.3389/fncom.2024.1345575

**Published:** 2024-01-31

**Authors:** Yiyan Zhang, Qin Li, Yi Xin

**Affiliations:** ^1^School of Intelligent Manufacturing, Qingdao Huanghai University, Qingdao, China; ^2^School of Life Science, Beijing Institute of Technology, Beijing, China

**Keywords:** algorithm applicability, data mining, dataset characteristic quantization, medical dataset, decision tree

## Abstract

With the vigorous development of data mining field, more and more algorithms have been proposed or improved. How to quickly select a data mining algorithm that is suitable for data sets in medical field is a challenge for some medical workers. The purpose of this paper is to study the comparative characteristics of the general medical data set and the general data sets in other fields, and find the applicability rules of the data mining algorithm suitable for the characteristics of the current research data set. The study quantified characteristics of the research data set with 26 indicators, including simple indicators, statistical indicators and information theory indicators. Eight machine learning algorithms with high maturity, low user involvement and strong family representation were selected as the base algorithms. The algorithm performances were evaluated by three aspects: prediction accuracy, running speed and memory consumption. By constructing decision tree and stepwise regression model to learn the above metadata, the algorithm applicability knowledge of medical data set is obtained. Through cross-verification, the accuracy of all the algorithm applicability prediction models is above 75%, which proves the validity and feasibility of the applicability knowledge.

## 1 Introduction

### 1.1 Background

With the development of data mining technology and interdisciplinary fields, more and more algorithms have been proposed and applied. With the development of science and the innovation of technology, hospital information system has been established and gradually popularized. The acquisition, storage and rapid transmission of large amounts of data are gradually realized, thus accumulating huge medical data resources. In the biomedical field, it is critical to translate the growing volume of biomedical data into meaningful and valuable information for practicing physicians. Traditional data analysis methods are mainly based on statistics. However, with the increasing of data sets, the wide application of multimedia storage media and object-oriented technology, the traditional statistical analysis methods are no longer enough to support the current data analysis needs. As a result, a series of new data analysis methods came into being, and data mining methods have been paid more and more attention and applied in the biomedical field. How to choose an algorithm, which is more suitable for the current task, from a large number of algorithms, is a problem to be solved in various research fields.

In this context, a novel and prospective research field - hybrid methods between metaheuristics and machine learning, has arisen. The novel research field successfully combines machine learning and swarm intelligence approaches and proved to be able to obtain outstanding results in different areas (Malakar et al., 2019; Bacanin et al., 2021, 2022; Zivkovic et al., 2022).

For medical workers without science and engineering background, it has become an urgent need to quickly choose a method suitable for current research data among many data mining algorithms. In view of the above problems, this paper adopts 8 data mining algorithms to construct models and evaluate results on different data sets according to research questions, obtain the applicability knowledge of algorithms, and provide empirical guidance for the selection of data mining algorithms. Aiming at the inconsistency of multiple evaluation indicators, this paper studied mapping knowledge from three aspects: prediction accuracy, modeling running time and memory occupancy requirements, which provided the possibility for users to choose according to the priority of research problems.

### 1.2 Related works

In 1976, Rice formally defined the conceptual model of algorithm selection, which consists of four parts: problem space, feature space, algorithm space and performance space (Rice, 1976). In order to make algorithm selection more targeted, Berrer introduced the concept of user preference into the algorithm evaluation system, enabling users to assign different weights to each evaluation index according to business characteristics, which is an important way for users to participate in the model selection process (Guoxun, 2013). Some early studies laid the foundation for meta-learning (Rendell and Cho, 1990; Aha, 1992; Schaffer, 2010; Jianshuang et al., 2017). Meta-learning, in simple terms, is learning about learning, that is, relearning on the basis of learning results (Brodley, 1995). Meta-learning studies how to learn from experience to enhance learning performance (Makmal et al., 2017). At present, researches on algorithm selection based on meta-learning ideas mainly focus on the description of dataset characteristics, the determination of meta-algorithms (Vilata and Drissi, 2002; Finn and Choi, 2017; Finn and Levine, 2017; Lee and Levine, 2018a,b) and the expansion and application of meta-learning to a specific problem (Doan, 2016; Li et al., 2017).

High-quality description of dataset characteristics can provide a reasonable explanation for the difference in algorithm performance, while few dataset characteristics were taken into account in early studies, which were expanded by two subsequent ESPRIT projects. (1) Comparative testing of statistical and logical learning(STATLOG) project (King et al., 1995): From 1991 to 1994, a large-scale project was carried out in Europe to compare classification algorithms. By applying different types of classification algorithms on different datasets from different fields, and comparing the performance of each algorithm, the relationship between algorithm performance and dataset characteristics was obtained, so as to provide empirical knowledge for algorithm selection. The STATLOG project selects 22 classification task datasets in the UCI database, 23 algorithms based on machine learning methods, such as statistics, rules, tree structure and neural network, and 16 dataset characteristics description indicators, such as mean, variance and information entropy. The accuracy of prediction is taken as the evaluation criterion. The C4.5 decision tree algorithm is used to generate rules applicable to data characteristics for each algorithm. The results of the STATLOG project show that no algorithm can perform optimally on all datasets, that confirms the No free lunch (NFL) theorem (Wolpert, 1996). The STATLOG project provides extremely valuable metadata that has been widely used in the field of meta-learning over the years. (2) A meta-learning assistant for providing user support in machine learning and data mining (METAL) project (Smith, 2008): From 1998 to 2001, based on the research results of the STATLOG project and the research progress of meta-learning, another algorithm selection research project was carried out in Europe, which mainly focused on algorithm selection in classification and regression problems. The METAL project selects a total of 53 classification task datasets from UCI database and other sources, 10 algorithms such as based on rules, decision trees, neural networks, instances and linear discrimination. The METAL project continues to use the 16 characteristic description indicators of datasets in STATLOG, and takes prediction accuracy and time performance as evaluation criteria. The computing performance of each algorithm is evaluated and sorted by 10-fold cross-validation.

After the two European ESPRIT projects, there is limited research on algorithm selection for general datasets without significant macro features. In 2000, Lim et al. selected 22 kinds of decision tree algorithms, 9 statistical algorithms and 2 neural network algorithms to run on 32 datasets respectively, and evaluated each algorithm in terms of classification accuracy, training time and number of leaf nodes in decision tree (Lim et al., 2000). In 2006, Ali and Smith conducted a large-scale algorithm selection study for classification problems. They selected 112 classification task datasets in the UCI database and 8 algorithms based on statistics, rules and neural networks. On the basis of STATLOG, they introduced statistical features from Matlab toolbox and other sources, such as the dispersion index and the maximum and minimum eigenvalues of covariance matrix, and expanded the characteristic description indicators of the dataset to 31. F-measure is added as evaluation criteria, and C4.5 decision tree algorithm is used to learn mapping rules to predict the optimal algorithm (Ali and Smith, 2006). For the first time, support vector machine (SVM) is included in the research scope, and the indicators of dataset characteristic description and algorithm evaluation are extended. Since 2014, some researchers focused on the integration of several basic classifiers (Cruz et al., 2015) or the overall workflow of some software (Nguyen and Kalousis, 2014; Soares, 2014). These studies only show the final result, which is equivalent to a black box for users, and the specific judgment process is unknown. For the specific field of supervised machine learning problems, Luo (2016) reviewed the literature on machine learning algorithms and automatic selection of hyperparameter values, and found that these methods have limitations in the context of biomedical data. Because the performance of machine learning algorithms is shown to be problem dependent (Heremans and Orshoven, 2015), it is recommended to compare different candidate algorithms in specific application environments. Some studies have been conducted in the fields of time series (Adhikari, 2015) and bioinformatics (Ding et al., 2014), which the data has significant temporal variation or high dimensional characteristics. Elmahgiubi (2016) developed a general meta-learning framework for automatic algorithm selection, applied to the selection problem of package classification algorithms and evaluated.

Algorithm selection should compare the performance of algorithms from multiple aspects. On the basis of some existing researches, the following three theorems have been widely recognized. (1) NFL theorem:Wolpert and Macerday proposed the NFL theorem for comparing two optimization algorithms to determine which one is better. However, the performance of the optimization algorithm is equivalent due to the mutual compensation of all possible functions. Specifically, it can be described as follows: For all optimization problems in a specific field, after m steps of iteration, the cumulative sum of all possibilities of algorithm A and algorithm B reaching the given value of the objective function is equal (David and Wolpert, 1997). NFL theorem shows that the algorithm is selected by the data, that is, the background of the problem. If we do not make any assumptions about the background of the problem, there is no universal optimal algorithm, so it is meaningless to study the universal optimal algorithm. (2) Occam’s razor principle (Warmuth, 1987): The principle states that “if it is not necessary, do not add entities,” that is, the “simple and effective principle.” The principle holds that for a given domain, the simplest explanation of a phenomenon is most likely to be correct, that is, for a given number of models with approximate goodness-of-fit, the more concise model should be chosen (Domingos, 2010). However, due to the simplicity and necessity of this principle is difficult to quantify in practice, this algorithm selection principle has not been widely promoted. (3) Minimum description length (MDL) principle (Rissanen, 1978): This principle was proposed by Rissanen (1978) from the perspective of information theory, and its basic idea is that for a given data set, the optimal compression of the data is the best hypothesis for the dataset. The MDL principle holds that the complexity of a model is the sum of the description length of the model itself and the encoding length of the data represented by the model (Barron et al., 1998). The principle is the formalization of Occam’s razor principle and one of the most practical branches of Kolmogorov complexity (Nannen, 2010). A highly complex hypothesis may accurately describe all the data, but lose generality at the same time. However, too simple description will miss a lot of data features, MDL principle is the compromise of the above two cases, avoids overfitting or underfitting of the model.

Ideally, we want to identify or design an algorithm that works best for all situations. However, both experimental results (Michie et al., 1994) and theoretical work (David, 1995) suggest that this is not possible. The choice of which algorithms to use depends on the dataset at hand, so a system that can provide recommendations for such choices would be very useful (Mitchell, 2003). By trying all the algorithms for this problem, we can narrow the algorithm recommendation problem down to a performance comparison problem. In practice, however, this is usually not feasible because there are too many algorithms to try, and some of them run slowly. This problem is exacerbated especially when dealing with large amounts of data, which often occurs in knowledge discovery in databases.

Many algorithm selection methods are limited to selecting a single algorithm or a small group of algorithms (Abdulrahman, 2017), that are expected to perform well on a given problem (Kalousis and Theoharis, 1999; Pfahringer and Bensusan, 2000; Todorovski, 2003). Brazdil et al. believe that the algorithm recommendation problem is more similar to the ranking task in nature, which is similar to the common ranking task in information retrieval and recommendation systems (Brazdil and Costa, 2003). In these tasks, it is not known in advance how many alternatives the user will actually consider. If the user’s preferred algorithm performs slightly less well than the one at the top of the ranking, the user can decide to stick with his favorite algorithm. If you have enough time and hardware conditions, you can try more algorithms. Since we do not know how many algorithms a user might actually want to choose, consider providing a ranking of all the algorithms. In 1994, Brazdil, Gama and Henery first used meta-learning algorithm recommendation to deal with sorting tasks (Brazdil, 1994). Later Nakhaeizadeh and Schnabl (1997), and later Keller et al. (2000), and Brazdil and Soares (2000) also adopted similar methods. In 2011, RBC Prudencio, MCPD Souto and TB Ludermir applied the ordering meta-learning method to the time series and gene expression data clustering field (Prudêncio et al., 2011). In 2017, Finn et al. introduced the theory of meta-learning in the fast adaptation study of deep networks (Finn and Levine, 2017).

The study of algorithm recommendation is the further improvement of the study of algorithm selection, and it is also the theoretical basis of the study of algorithm applicability in this paper.

Medical data has different characteristics from other data. The theoretical framework for the applicability study of medical data mining algorithm proposed and constructed in this paper can provide more targeted empirical knowledge on algorithm selection for medical research compared with previous studies. The algorithm applicability knowledge base constructed in this paper solves the problem of lack of empirical knowledge of data mining algorithms in medical research, and provides theoretical guidance for users to choose suitable algorithms.

## 2 Materials and methods

### 2.1 Base dataset

In the selection of datasets, this paper follows the principles of universality, openness and less intervention, and uses the machine learning database of University of California Irvine (UCI) as the source of the base dataset. The UCI database is a database used by the machine learning community for empirical analysis of machine learning algorithms, and it is a collection of data that covers domain theory data as well as data generated by data generators. Since inception in 1987 by David Aha and others, the UCI database has been used by students, teachers, and researchers around the world as the primary source of machine learning datasets. At present, the UCI database has reached more than 1,000 citations, making it one of the top 100 most cited in computer science. According to the dataset range studied in this paper, that is, open data sets aiming at classification that can be converted into structured data through simple or slightly complex operations, open datasets included in UCI database are selected. One hundred and thirty-eight independent datasets from 335 UCI datasets were included in the study.

### 2.2 Data preprocessing

The datasets in the UCI database come from various industries, and a considerable part of them are shared raw data. The data collection and storage software used by the sharers are not the same, so there are some differences in data formats. The quality of data is the basic guarantee of data analysis, and only high-quality data can obtain high-quality analysis results. Therefore, this paper conducted data preprocessing on 138 selected datasets in order to carry out characteristic quantization and subsequent algorithm applicability research. Since the purpose of this paper is to study the characteristics of universal medical datasets compared with general datasets in other fields, the principle of “only necessary preprocessing without affecting the basic characteristics of data” is adhered to in the data preprocessing stage. Specifically, that is to simulate the preliminary data preprocessing carried out by the researchers after obtaining the original data for the current research scheme. Data preprocessing in the study mainly includes the following aspects:

#### 2.2.1 Deficient data

In the process of data acquisition, many reasons may cause the incompleteness of collected data. For datasets that lack a column name, define the column name to clarify the meaning of the attribute. Since medical data involves different individuals, and individual differences exist among patients, it is easy to introduce greater errors if the missing values are filled by mean, median, chain equation and other methods hastily. Therefore, data samples containing missing values are removed in this paper to ensure the integrity of each analysis sample. At the same time, in order to avoid a large reduction in the sample size of the dataset after excessive removal of missing values of a variable, this paper with a limit of 30%, removes attributes with missing values exceeding 30%. Because some attributes in the dataset have more missing values, if the samples with missing values are directly removed, the sample size of the dataset will be greatly reduced. Therefore, the threshold of 30% is set in this study. When the missing value ratio of an attribute is greater than this threshold, the attribute will be removed.

#### 2.2.2 Inconsistent data

In the process of data recording and collection, there may be inconsistent presentation, spelling errors and other problems resulting in inconsistent data. In this paper, by comparing with the description of the dataset, the inconsistent data that can be clearly judged are normalized, the uncertain differences are retained and multi-party verification is carried out, and the sample data is removed if there is no confirmed information to reduce noise.

#### 2.2.3 Data integration

Different data collection scenarios and storage media will cause the collected data to be dispersed in different data files, showing the characteristics of phased and distributed storage. In this case, the data of different data sources need to be associated and integrated through data integration operations, and stored in a unified data set.

After data preprocessing, a total of 293 sub datasets of 138 independent datasets were included in this study.

### 2.3 Dataset characteristic metadata

By focusing on the analysis and comparison of the calculation indicators adopted by the two European Spirit projects - STATLOG and METAL, and combining the research purpose and needs of this study, this paper adopts 26 indicators to quantify the characteristics of the research datasets. These 26 quantitative indicators can be divided into three categories: simple indicators, statistical indicators and information theory indicators.

#### 2.3.1 Simple indicators

1. Number of variables (P).
2. Sample size (N).
3. Number of categories (N_class).
4. Ratio of largest class (R_largest).
5. Ratio of least class (R_least).
6. Ratio of binary variable (R_binary).
7. Ratio of discrete variable (R_discrete).
8. Ratio of continuous variable (R_continuous).
9. Ratio of missing values (R_missing).

#### 2.3.2 Statistical indicators

1. Geometric mean (Geomean).
2. Harmonic mean (Harmean).
3. Trim mean (Trimean).
4. Percentile (Prctile).
5. Mean absolute deviation (MAD).
6. Variance (Var).
7. Standard deviation (Std).
8. Mean of absolute correlation coefficient (MAr).
9. Interquartile range (IQR).
10. Index of dispersion (D).
11. Skewness.
12. Kurtosis.

#### 2.3.3 Informational indicators

1. Mean entropy of attribute variables (ME_V).
2. Entropy of class (E_C).
3. Mean mutual entropy of class and attribute variables (MME_CV).
4. Equivalent number of variables (ENV): The ratio of E_C to MME_CV.
5. Noise-signal ratio (NSR).

### 2.4 Base algorithm selection

Classification, as one of the most important techniques in data mining, has a wide applicable range, and many classification algorithms have been proposed so far. According to the learning characteristics of each algorithm, data mining classification algorithms can be divided into the following four categories: classification algorithm based on tree, classification algorithm based on neural network, classification algorithm based on Bayes, and classification algorithm based on statistics. In recent years, on the basis of statistical learning theory, support vector machine (SVM) have developed vigorously, showed unique advantages in solving small sample, nonlinear and high-dimensional pattern recognition problems, and received attention and promotion from scholars in multiple fields. In addition, rough set theory, fuzzy set theory, genetic algorithm and ensemble learning methods are introduced into the classification task.

In the study, the following three selection criteria for alternative base algorithms are formulated:

1. High maturity in theory and practice;
2. Less user involvement in the design stage;
3. Strong family representation.

According to the above three criteria, this paper filters many data mining algorithms for aiming at classification task. This paper selects five classification algorithms among the ten classic algorithms: k nearest neighbor (kNN) algorithm, decision tree C4.5 (C4.5) algorithm, support vector machine (SVM) algorithm, naive bayes (NB) algorithm, AdaBoost (AB) algorithm, and the increasingly popular - random forest (RF) algorithm, the representative of neural network algorithm - backpropagation network (BP), and logistic regression (LR), which is commonly used in medical research. The above 8 algorithms are used as the alternative base algorithm in this paper.

### 2.5 Algorithm performance metadata

In the process of algorithm applicability research, algorithm performance evaluation is an essential component. In the field of machine learning, the commonly used algorithms performance evaluation indexes include: accuracy rate, true positive rate, true negative rate, recall rate, average absolute error, Area under the ROC curve (AUC), Akaike information criterion (AIC), running time, interpretability, etc. For different data mining methods, there are specific evaluation indexes.

The evaluation of the classification methods is mainly based on the following five items:

1. Accuracy of prediction: the proportion of correct classification in sample data;
2. Running speed: the time of model construction and classification using the model. Since the time required to generate the model accounts for most of the total time, the model construction time is mainly used as the measurement standard of the speed of the classification method in the experiment;
3. Robustness: The ability of the model to accurately predict data with noise or missing values;
4. Processable data volume: The ability to effectively construct a model in the face of a large amount of data, mainly referring to the ability to solve the problem of resident disk data;
5. Interpretability: The level at which a model can be understood.

In the field of medical research, sensitivity, specificity and accuracy are often used to evaluate predictive models constructed in a particular study. Sensitivity is the proportion of individuals with actual disease who are accurately judged to be true positive, that is, the true positive rate described above. Specificity is the proportion of individuals who are not actually sick that are accurately judged to be true negative, i.e., the true negative rate and recall rate described above.

By focusing on the analysis and comparison of the calculation indicators adopted by the two European Spirit projects - STATLOG and METAL, and combining the research purpose and needs of this study, this paper mainly evaluates each alternative base algorithm in three aspects, the prediction accuracy, running speed and memory consumption.

#### 2.5.1 Prediction accuracy

The accuracy ( Acc ) of training set and test set, as well as the analog expansion of sensitivity and specificity, are used as the evaluation indexes for the prediction accuracy of each alternative base algorithm.

In this paper, the analogy of sensitivity and specificity can be briefly described as calculating the correct prediction rate of the class with the most and least samples in the target variable, respectively denoted as S_largest and S_least . The calculation formulas are shown in (1) and (2).

(1) $$\begin{array}{l} S\_largest=Thenumberofsamplescorrectlypredictedin\\ \;\;\;\;\;\;\;\;\;\;\;\;\;\;\;\;\;\;\;\;\;\;\;\;\;\;\;\;\;\;\;thecategorywiththelargestsamplesize\\ \;\;\;\;\;\;\;\;\;\;\;\;\;\;\;\;\;\;\;\;\;\;\;\;\;\;\;\;\;/Theactualnumberofsamplesinthecategory\\ \;\;\;\;\;\;\;\;\;\;\;\;\;\;\;\;\;\;\;\;\;\;\;\;\;\;\;\;withthelargestsamplesize\times 100\% \end{array}$$

(2) $$\begin{array}{l} S\_least=Thenumberofsamplescorrectlypredictedinthe\\ \;\;\;\;\;\;\;\;\;\;\;\;\;\;\;\;\;\;\;\;\;\;\;\;\;\;\;categorywiththesmallestsamplesize\\ \;\;\;\;\;\;\;\;\;\;\;\;\;\;\;\;\;\;\;\;\;\;\;\;\;\;/Theactualnumberofsamplesinthecategory\\ \;\;\;\;\;\;\;\;\;\;\;\;\;\;\;\;\;\;\;\;\;\;\;\;\;\;withthesmallestsamplesize\times 100\% \end{array}$$

#### 2.5.2 Running speed

The modeling time of 8 alternative base algorithms on each base dataset is monitored and collected as an evaluation indicator. Since each algorithm will produce an order of magnitude difference in the dataset with different characteristics, the logarithmic operation of the modeling time of each algorithm is carried out in order to carry out comparative analysis.

#### 2.5.3 Memory consumption

Monitor and collect the memory occupation of the prediction model built by 8 alternative base algorithms on each base dataset as an evaluation indicator. Considering that each algorithm will produce an order of magnitude difference in the dataset with different characteristics, the logarithmic operation of the memory usage of each algorithm is carried out for comparative analysis.

For different research objectives and programs, the focus of researchers may be different. For the diagnosis of a rare disease, researchers are more concerned about the identification and screening rate of this minority group of people with the disease, that is, the above S_least value need to meet the acceptable threshold. For the diagnosis or prediction of the development of some emergency conditions, such as judging whether a patient with chest pain is an acute myocardial infarction or a patient in need of timely intervention in the emergency room, the prediction model to be used at this time has high requirements on the prediction accuracy and time, that is, the performance evaluation algorithm indicators mentioned above need to be considered comprehensively.

### 2.6 Algorithm applicability evaluation

Because several algorithms reach the optimal level on some datasets at the same time, the optimal algorithm result is the combination of several algorithms. The number of these combinations can be reduced by combining the prediction accuracy evaluation with the runtime and memory usage, respectively. However, due to the differences in dataset characteristics that affect the running time and memory usage, this method has some defects. Considering the ratio between the number of datasets included in this paper and the combined results, in order to ensure the accuracy and generalization of the algorithm applicability knowledge, we decided to discretized the ranking of prediction accuracy of each algorithm on each dataset, that is, the top three algorithms are labeled as recommended algorithm (Y), and the fourth and fifth algorithms are labeled as medium (M), ranking sixth through eighth and modeling failures are marked as not recommended (No).

Due to the 34 discrete variable datasets included in the study, limited by the amount of data, they are not suitable for modeling learning features. Therefore, this paper only conducts modeling learning on mixed variable datasets and continuous variable datasets to evaluate the algorithm applicability on different characteristic datasets.

## 3 Results

### 3.1 Preliminary statistical results

In 293 UCI data subsets included in the study, modeling failures occurred in all eight algorithms. Among them, the main reason for LR algorithm modeling failure is that the dimension is too high or the number of weight coefficients contained in the discrete variable exceeds the maximum threshold allowed by the algorithm, resulting in modeling failure. The main reason for AB algorithm modeling failure is memory overflow, that is, the memory required for modeling exceeds the upper limit allocated by the system. The main reason for RF algorithm modeling failure is discrete variables include too many categories exceeding the upper limit and memory overflow. The main reason for BP algorithm modeling failure is basically the same with LR. The modeling success rate of the eight algorithms is shown in Table 1.

**Table 1:** Summary of modeling completed by 8 algorithms.

Algorithm	Mixed variable datasets		Discrete variable datasets		Continuous variable datasets		Total failed
	Completed	Failed	Completed	Failed	Completed	Failed	
LR	93	9	29	5	138	19	33
C4.5	101	1	34	0	157	0	1
SVM	101	1	33	1	157	0	2
AB	96	6	32	2	153	4	12
kNN	101	1	33	1	157	0	2
NB	102	0	34	0	157	0	0
RF	95	7	33	1	152	5	13
BP	68	34	25	9	133	24	67

As can be seen from Table 1 that the BP algorithm modeling failure rate is relatively high, 22.87%. Preliminary analysis, the number of weight coefficients exceeded the maximum threshold allowed by the algorithm due to too many categories of discrete variables. Further analysis and discussion will be conducted in accordance with the specific characteristics of the dataset.

**Figure 1:**
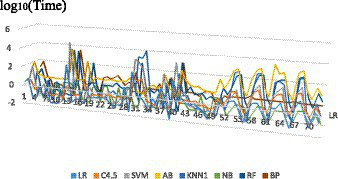
Modeling time of 8 algorithms on different datasets.

Since the learning and modeling time of the eight algorithms on different datasets presents an order of magnitude difference, the learning and modeling time result values after logarithmic are compared in this paper, and the scatter diagram is shown in Figure 1.

The number on X axis corresponds to the serial number of the research dataset. As can be seen from Figure 1 that the same algorithm has different learning and modeling time on datasets with different characteristics. The overall trend shows that the modeling time of NB algorithm is the shortest on most datasets, while the modeling time of ensemble method AB is significantly several orders of magnitude higher. Dataset characteristics that affect modeling time will be further discussed and analyzed later.

**Figure 2:**
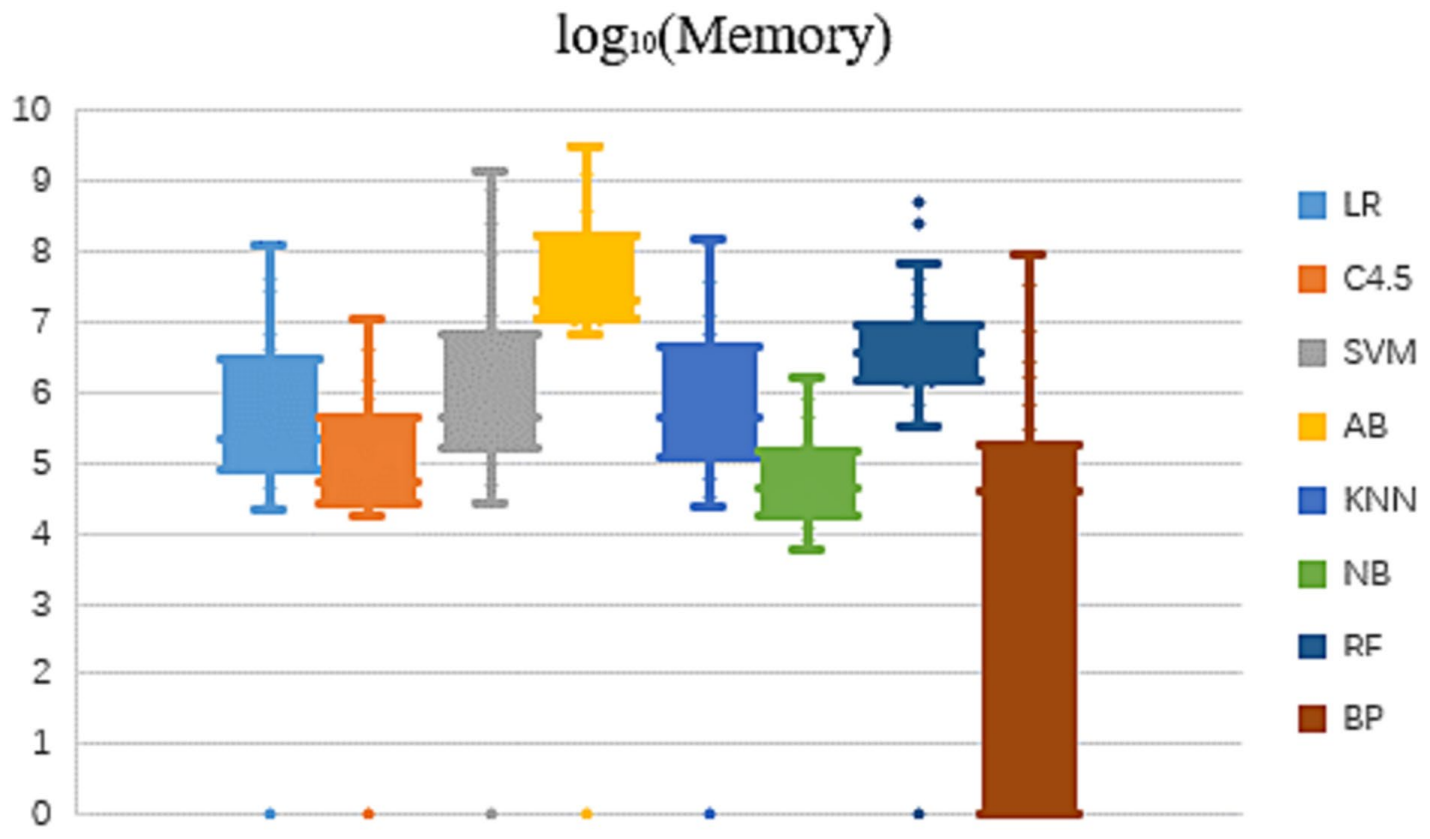
Modeling memory usage of 8 algorithms on different datasets.

In view of the fact that the memory usage of the eight algorithms in learning and modeling on different datasets also presents an order of magnitude difference, this paper compares the memory occupation result values after logarithmic, as shown in Figure 2.

As can be seen from Figure 2 that the memory occupied by the same algorithm is different to some extent, when learning and modeling on datasets with different characteristics. The overall trend shows that on most datasets, NB algorithm requires the smallest amount of memory for modeling, followed by C4.5 algorithm, while RF and AB two ensemble methods have significantly higher memory consumption of several orders of magnitude. Dataset characteristics that affect memory usage will be further analyzed and summarized in subsequent studies.

Because the number of the discrete variable dataset is small, the modeling analysis is not used, but the chi-square test analysis of R*C contingency table is carried out. The recommendation of the 8 algorithms on the datasets in different fields was sorted into contingency tables, respectively. Taking the LR algorithm as an example, as shown in Table 2, the differences between groups were compared by the $\chi^{2}$ values calculated according to formula (3). Similarly, contingency table analysis was performed on the other 7 base algorithms to explore the applicability of each algorithm on the dataset in the biomedical field. The contingency table analysis results of whether there are differences in data domain among LR, C4.5, SVM, AB, kNN, NB, RF and BP 8 base algorithms are shown in Table 3.

(3) $$\chi^{2}=n\left(\overset{\begin{array}{c} i=R\\ j=C \end{array}}{\underset{i,j=1}{\sum }}\frac{A_{ij}^{2}}{n_{R_{i}}n_{C_{j}}}-1\right)$$

**Table 2:** The recommended usage of LR algorithm on different domain datasets – discrete variable datasets.

LR	Y	M	No
Medical	1	2	2
Biology	3	3	2
General	3	5	13

**Table 3:** R*C contingency table analysis results of 8 algorithms – discrete variable datasets.

Algorithm	$\chi^{2}$	Difference between groups
LR	3.8052	No
C4.5	2.9952	No
SVM	2.1799	No
AB	4.2213	No
kNN	3.8980	No
NB	11.2935	Yes
RF	7.9518	No
BP	7.5466	No

In the formula, $A_{ij}$ is the actual frequency of each cell in the contingency table, and $n_{R_{i}}$ and $n_{C_{j}}$ are the combined counts of row i and column j corresponding to $A_{ij}$ .

As can be seen from Table 3, there are differences in the recommendation of NB algorithm on datasets in the medical, biological and general fields. By referring to the occurrence table of NB algorithm, it can be found that the recommendation rate of NB algorithm on datasets in the medical and biological fields is relatively high, which is 60.0 and 50.0% respectively, while the recommendation rate on datasets in the general field is only 4.8%.

### 3.2 Predictive accuracy modeling analysis

Through the above exploratory analysis, we have a preliminary understanding of the algorithm applicability. In order to further discover the hidden feature knowledge in the algorithm applicability, this paper uses stepwise regression and decision tree C4.5 algorithm to build a model, so as to find the features and rules that need to be further analyzed and discussed in the previous exploratory statistical analysis.

#### 3.2.1 Mixed variable datasets

With “whether to recommend” as the target variable and 26 quantization characteristics of datasets as attribute variables, a stepwise regression model was constructed to obtain dataset characteristics related to the applicability of LR, C4.5, SVM, AB, kNN, NB, RF and BP 8 algorithms, as shown in Table 4.

**Table 4:** Summary of dataset characteristics related to the applicability of 8 algorithms – mixed variable datasets.

Dataset characteristic	LR	C4.5	SVM	AB	kNN	NB	RF	BP
P	√	√	√		√		√	√
N		√	√	√	√	√	√	√
N_class		√	√		√		√	
R_largest			√	√	√		√	
R_least	√	√	√		√	√		√
R_binary		√		√	√	√		
R_discrete	√			√	√	√	√	
R_continous	√				√	√		√
Geomean		√	√		√	√	√	
Harmean	√		√	√	√		√	√
MAD	√	√			√	√	√	√
Var			√		√			
Std	√		√			√		√
MAr		√	√	√	√	√	√	
IQR			√	√				√
D	√		√	√	√		√	√
Skewness	√		√	√	√	√		
Kurtosis		√	√	√		√	√	√
Trimean	√		√	√	√	√		
Percentile		√	√		√	√		√
ME_V		√		√	√	√		√
E_C	√				√	√		√
MME_CV	√	√		√	√		√	
ENV	√		√	√	√	√	√	√
NSR	√		√	√	√			√
Field label				√		√		√

In Table 4, “√” indicates that there is a statistically significant correlation between an algorithm and a dataset characteristic after stepwise regression screening.

With “whether to recommend” as the target variable and the 26 quantization characteristics of datasets as attribute variables, a decision tree model was constructed using the C4.5 algorithm. The applicability judgment decision trees of 8 algorithms on the obtained mixed variable data set are built. According to the decision trees, the applicability of 8 algorithms on mixed variable datasets can be judged and predicted.

#### 3.2.2 Continuous variable datasets

In Table 5, after stepwise regression screening, there is a statistically significant correlation between an algorithm and the characteristics of a dataset, which is represented by “√”.

**Table 5:** Summary of dataset characteristics related to the applicability of 8 algorithms – continuous variable datasets.

Dataset characteristic	LR	C4.5	SVM	AB	kNN	NB	RF	BP
P	√	√	√	√	√	√	√	
N	√	√	√	√	√		√	√
N_class	√	√	√			√		√
R_largest	√		√	√	√	√	√	
R_least	√			√				√
Geomean			√	√	√	√		√
Harmean			√			√		√
MAD	√		√			√	√	√
Var	√	√	√	√	√	√	√	
Std		√		√	√	√	√	
MAr	√		√	√	√	√	√	
IQR	√		√			√	√	√
D		√				√	√	√
Skewness	√	√	√	√	√	√	√	√
Kurtosis	√	√	√				√	√
Trimean	√	√	√			√		
Percentile	√		√			√	√	√
E_C	√		√			√		
Field label		√	√			√	√	√

With “recommend or not” as the target variable and 26 data sets quantization characteristics as attribute variables, a decision tree model is constructed using C4.5 algorithm. The applicability judgment decision tree of 8 algorithms obtained on continuous variable datasets are built. According to these decision trees, the applicability of 8 algorithms on continuous variable datasets can be judged and predicted. Through the validation on the training set and test set of the algorithm applicability metadata, both the decision tree judgment model and the stepwise regression judgment model reached the accuracy of more than 75%.

### 3.3 Running time modeling analysis

Since the learning and modeling time of the eight algorithms on different datasets presents an order of magnitude difference, this paper calculates the running time logarithmic value, and then construct a model to perform magnitude prediction.

Associated the running time of the algorithm with the dataset characteristics, and analyzed the integrated metadata set. Taking “log10(Time)” as the target variable and 26 quantized dataset characteristics as the attribute variables. Firstly, the correlation between the target variable and the attribute variable is calculated, and the attribute variable with the absolute value of the correlation coefficient greater than 0.3 is taken as the correlation variable and included in the next modeling analysis. The model was constructed by stepwise regression, and the running time order prediction formulas of LR, C4.5, SVM, AB, kNN, NB, RF and BP 8 algorithms on the three categories datasets were obtained respectively, as shown in formulas (4)–(27).

#### 3.3.1 Mixed variable datasets

The running time magnitude prediction formula of LR algorithm on mixed variable datasets is shown in formula (4).

(4) $$\begin{array}{l} \log_{10}{\left(Time\right)}_{LR}=-0.0034-3.948^{*}R\_least\\ \;\;\;\;\;\;\;\;\;\;\;\;\;\;\;\;\;\;\;\;\;\;\;\;\;\;\;\;\;\;\;\;\;\;\;\;\;\;\;\;\;\;+0.000002^{*}N-0.0149^{*}\mathrm{Harmean} \end{array}$$

The running time magnitude prediction formula of C4.5 algorithm on mixed variable datasets is shown in formula (5).

(5) $$\begin{array}{l} log_{10}{\left(Time\right)}_{C4.5}=0.607+0.000001*N\\ \;\;\;\;\;\;\;\;\;\;\;\;\;\;\;\;\;\;\;\;\;\;\;\;\;\;\;\;\;\;\;\;\;\;\;\;\;\;\;\;\;\;\;\;\;-2.349*R\_least-0.7297*E\_C\\ \;\;\;\;\;\;\;\;\;\;\;\;\;\;\;\;\;\;\;\;\;\;\;\;\;\;\;\;\;\;\;\;\;\;\;\;\;\;\;\;\;\;\;\;\;-0.011*Harmean \end{array}$$

The running time magnitude prediction formula of SVM algorithm on mixed variable datasets is shown in formula (6).

(6) $$\begin{array}{l} log_{10}{\left(Time\right)}_{SVM}=-0.4845+0.000003*N\;-2.263*R\_least\\ \;\;\;\;\;\;\;\;\;\;\;\;\;\;\;\;\;\;\;\;\;\;\;\;\;\;\;\;\;\;\;\;\;\;\;\;\;\;\;\;\;\;\;\;\;\;-0.0206*Harmean+1.047*R\_largest \end{array}$$

The running time magnitude prediction formula of AB algorithm on mixed variable datasets is shown in formula (7).

(7) $$\begin{array}{l} \log_{10}{\left(Time\right)}_{AB}=2.171+0.000001^{*}N\\ \;\;\;\;\;\;\;\;\;\;\;\;\;\;\;\;\;\;\;\;\;\;\;\;\;\;\;\;\;\;\;\;\;\;\;\;\;\;\;\;\;-2.358^{*}R\_least-1.205^{*}E\_C \end{array}$$

The running time magnitude prediction formula of kNN algorithm on mixed variable datasets is shown in formula (8).

(8) $$\begin{array}{l} \log_{10}{\left(Time\right)}_{kNN}=0.9135+0.000005^{*}N\\ \;\;\;\;\;\;\;\;\;\;\;\;\;\;\;\;\;\;\;\;\;\;\;\;\;\;\;\;\;\;\;\;\;\;\;\;\;\;\;\;\;\;\;\;\;-4.344^{*}R\_least-2.054^{*}E\_C \end{array}$$

The running time magnitude prediction formula of NB algorithm on mixed variable datasets is shown in formula (9).

(9) $$\begin{array}{l} log_{10}{\left(Time\right)}_{NB}=-0.6855+0.000002*N-0.5441*R\_binary\\ \;\;\;\;\;\;\;\;\;\;\;\;\;\;\;\;\;\;\;\;\;\;\;\;\;\;\;\;\;\;\;\;\;\;\;\;\;\;\;\;\;\;-1.241*ME\_V-1.228*R\_least\\ \;\;\;\;\;\;\;\;\;\;\;\;\;\;\;\;\;\;\;\;\;\;\;\;\;\;\;\;\;\;\;\;\;\;\;\;\;\;\;\;\;\;-0.528*E\_C+0.0013*P \end{array}$$

The running time magnitude prediction formula of RF algorithm on mixed variable datasets is shown in formula (10).

(10) $$\begin{array}{l} \log_{10}{\left(Time\right)}_{RF}=1.395+0.000002^{*}N-3.898^{*}R\_least\\ \;\;\;\;\;\;\;\;\;\;\;\;\;\;\;\;\;\;\;\;\;\;\;\;\;\;\;\;\;\;\;\;\;\;\;\;\;\;\;\;\;\;\;-1.439^{*}E\_C-0.0167^{*}Harmean \end{array}$$

The running time magnitude prediction formula of BP algorithm on mixed variable datasets is shown in formula (11).

(11) $$\begin{array}{l} \log_{10}{\left(Time\right)}_{BP}=-4.285+0.000003^{*}N-4.976^{*}R\_l\arg est\\ \;\;\;\;\;\;\;\;\;\;\;\;\;\;\;\;\;\;\;\;\;\;\;\;\;\;\;\;\;\;\;\;\;\;\;\;\;\;\;\;\;\;\;+3.845^{*}E\_C-0.008^{*}Harmean \end{array}$$

#### 3.3.2 Discrete variable datasets

The running time magnitude prediction formula of LR algorithm on discrete variable datasets is shown in formula (12).

(12) $$\begin{array}{l} \log_{10}{\left(Time\right)}_{LR}=-1.419+0.00007^{*}N+0.0526^{*}N\_\mathrm{class}\\ \;\;\;\;\;\;\;\;\;\;\;\;\;\;\;\;\;\;\;\;\;\;\;\;\;\;\;\;\;\;\;\;\;\;\;\;\;\;\;\;\;\;+0.0074^{*}\mathrm{ENV} \end{array}$$

The running time magnitude prediction formula of C4.5 algorithm on discrete variable datasets is shown in formula (13).

(13) $$\begin{array}{l} \log_{10}{\left(Time\right)}_{C4.5}=-0.8812+0.00003^{*}N\\ \;\;\;\;\;\;\;\;\;\;\;\;\;\;\;\;\;\;\;\;\;\;\;\;\;\;\;\;\;\;\;\;\;\;\;\;\;\;\;\;\;\;\;\;\;+0.0015^{*}P+0.0097^{*}N\_class \end{array}$$

The running time magnitude prediction formula of SVM algorithm on discrete variable datasets is shown in formula (14).

(14) $$\begin{array}{l} \log_{10}{\left(Time\right)}_{SVM}=-1.359+0.00009^{*}N\\ \;\;\;\;\;\;\;\;\;\;\;\;\;\;\;\;\;\;\;\;\;\;\;\;\;\;\;\;\;\;\;\;\;\;\;\;\;\;\;\;\;\;\;\;\;+0.0087^{*}ENV+0.8517^{*}E\_C \end{array}$$

The running time magnitude prediction formula of AB algorithm on discrete variable datasets is shown in formula (15).

(15) $$\begin{array}{l} \log_{10}{\left(Time\right)}_{AB}=0.8643+0.00004^{*}N+0.003^{*}ENV\\ \;\;\;\;\;\;\;\;\;\;\;\;\;\;\;\;\;\;\;\;\;\;\;\;\;\;\;\;\;\;\;\;\;\;\;\;\;\;\;\;\;\;+0.0183^{*}N\_class-0.4194^{*}E\_C\\ \;\;\;\;\;\;\;\;\;\;\;\;\;\;\;\;\;\;\;\;\;\;\;\;\;\;\;\;\;\;\;\;\;\;\;\;\;\;\;\;\;\;\;+0.0019^{*}P \end{array}$$

The running time magnitude prediction formula of kNN algorithm on discrete variable datasets is shown in formula (16).

(16) $$\log_{10}{\left(Time\right)}_{kNN}=-1.064+0.00006^{*}N$$

The running time magnitude prediction formula of NB algorithm on discrete variable datasets is shown in formula (17).

(17) $$\begin{array}{l} \log_{10}{\left(Time\right)}_{NB}=-1.983+0.0017^{*}P+0.1835^{*}R\_\mathrm{binary}\\ \;\;\;\;\;\;\;\;\;\;\;\;\;\;\;\;\;\;\;\;\;\;\;\;\;\;\;\;\;\;\;\;\;\;\;\;\;\;\;\;\;\;+0.000018^{*}N \end{array}$$

The running time magnitude prediction formula of RF algorithm on discrete variable datasets is shown in formula (18).

(18) $$\log_{10}{\left(Time\right)}_{RF}=-1.641+0.00005^{*}N+1.566^{*}E\_C$$

The running time magnitude prediction formula of BP algorithm on discrete variable datasets is shown in formula (19).

(19) $$\log_{10}{\left(Time\right)}_{BP}=-0.7675+0.00006^{*}N+0.0549^{*}N\_class$$

#### 3.3.3 Continuous variable datasets

The running time magnitude prediction formula of LR algorithm on continuous variable datasets is shown in formula (20).

(20) $$\begin{array}{l} \log_{10}{\left(Time\right)}_{LR}=-0.5703+0.000009^{*}N-1.897^{*}R\_least\\ \;\;\;\;\;\;\;\;\;\;\;\;\;\;\;\;\;\;\;\;\;\;\;\;\;\;\;\;\;\;\;\;\;\;\;\;\;\;\;\;\;\;+0.0322^{*}N\_class\\ \;\;\;\;\;\;\;\;\;\;\;\;\;\;\;\;\;\;\;\;\;\;\;\;\;\;\;\;\;\;\;\;\;\;\;\;\;\;\;\;\;\;+0.0000008^{*}Geomean \end{array}$$

The running time magnitude prediction formula of C4.5 algorithm on continuous variable datasets is shown in formula (21).

(21) $$\log_{10}{\left(Time\right)}_{C4.5}=-0.2581+0.000006^{*}N-0.7944^{*}R\_least$$

The running time magnitude prediction formula of SVM algorithm on continuous variable datasets is shown in formula (22).

(22) $$\begin{array}{l} \log_{10}{\left(Time\right)}_{SVM}=0.0992+0.00001^{*}N+0.000001^{*}Geomean\\ \;\;\;\;\;\;\;\;\;\;\;\;\;\;\;\;\;\;\;\;\;\;\;\;\;\;\;\;\;\;\;\;\;\;\;\;\;\;\;\;\;\;\;\;\;-4.144^{*}MAr-1.998^{*}R\_least \end{array}$$

The running time magnitude prediction formula of AB algorithm on continuous variable datasets is shown in formula (23).

(23) $$\begin{array}{l} \log_{10}{\left(Time\right)}_{AB}=1.221+0.000008^{*}N+0.0000003^{*}Geomean\\ \;\;\;\;\;\;\;\;\;\;\;\;\;\;\;\;\;\;\;\;\;\;\;\;\;\;\;\;\;\;\;\;\;\;\;\;\;\;\;\;\;\;-0.5567^{*}R\_least \end{array}$$

The running time magnitude prediction formula of kNN algorithm on continuous variable datasets is shown in formula (24).

(24) $$\begin{array}{l} \log_{10}{\left(Time\right)}_{kNN}=-0.3235+0.00001^{*}N+0.0000008^{*}Geomean\\ \;\;\;\;\;\;\;\;\;\;\;\;\;\;\;\;\;\;\;\;\;\;\;\;\;\;\;\;\;\;\;\;\;\;\;\;\;\;\;\;\;\;\;\;\;\;-3.446^{*}MAr-1.569^{*}R\_least \end{array}$$

The running time magnitude prediction formula of NB algorithm on continuous variable datasets is shown in formula (25).

(25) $$\log_{10}{\left(Time\right)}_{NB}=-1.578+0.000004^{*}N$$

The running time magnitude prediction formula of RF algorithm on continuous variable datasets is shown in formula (26).

(26) $$\begin{array}{l} \log_{10}{\left(Time\right)}_{RF}=0.1164+0.000009^{*}N-1.513^{*}R\_least\\ \;\;\;\;\;\;\;\;\;\;\;\;\;\;\;\;\;\;\;\;\;\;\;\;\;\;\;\;\;\;\;\;\;\;\;\;\;\;\;\;\;+0.0000006^{*}Geomean \end{array}$$

The running time magnitude prediction formula of BP algorithm on continuous variable datasets is shown in formula (27).

(27) $$\begin{array}{l} \log_{10}{\left(Time\right)}_{BP}=0.7947+0.00001^{*}N-2.448^{*}R\_least\\ \;\;\;\;\;\;\;\;\;\;\;\;\;\;\;\;\;\;\;\;\;\;\;\;\;\;\;\;\;\;\;\;\;\;\;\;\;\;\;\;\;\;-3.449^{*}MAr \end{array}$$

### 3.4 Memory requirement modeling analysis

The memory usage of algorithms during running is related to the inherent characteristics of the dataset, and there will be differences of orders of magnitude among each algorithm on the same dataset. Therefore, logarithmic operation is carried out on the memory occupation of the learning and modeling process of each algorithm for comparative analysis and prediction.

The memory usage of the algorithm is associated with the dataset characteristics, and the integrated metadata set is learned and analyzed, with “log10(Memory)” as the target variable and 26 quantization characteristics of the dataset as the attribute variable. Firstly, the correlation between the target variable and the attribute variable is calculated, and the attribute variable with the absolute value of the correlation coefficient greater than 0.3 is taken as the correlation variable and included in the next modeling analysis. The model was constructed by stepwise regression, and the prediction formulas of the memory usage level of LR, C4.5, SVM, AB, kNN, NB, RF and BP 8 algorithms on the three categories datasets were obtained, as shown in formulas (28)–(51).

#### 3.4.1 Mixed variable datasets

The memory usage level prediction formula of LR algorithm on mixed variable datasets is shown in formula (28).

(28) $$\begin{array}{l} \log_{10}{\left(Memory\right)}_{LR}=5.055+0.000003^{*}N-1.556^{*}R\_least\\ \;\;\;\;\;\;\;\;\;\;\;\;\;\;\;\;\;\;\;\;\;\;\;\;\;\;\;\;\;\;\;\;\;\;\;\;\;\;\;\;\;\;\;\;\;\;\;\;\;\;\;+0.7283^{*}R\_largest+0.554^{*}R\_binary \end{array}$$

The memory usage level prediction formula of C4.5 algorithm on mixed variable datasets is shown in formula (29).

(29) $$\begin{array}{l} \log_{10}{\left(Memory\right)}_{C4.5}=3.354+0.0021^{*}P+0.000002^{*}N\\ \;\;\;\;\;\;\;\;\;\;\;\;\;\;\;\;\;\;\;\;\;\;\;\;\;\;\;\;\;\;\;\;\;\;\;\;\;\;\;\;\;\;\;\;\;\;\;\;\;\;\;\;\;\;+1.574^{*}R\_largest+0.9187^{*}E\_C \end{array}$$

The memory usage level prediction formula of SVM algorithm on mixed variable datasets is shown in formula (30).

(30) $$\begin{array}{l} \log_{10}{\left(Memory\right)}_{SVM}=6.427+0.000002^{*}N-2.149^{*}R\_least\\ \;\;\;\;\;\;\;\;\;\;\;\;\;\;\;\;\;\;\;\;\;\;\;\;\;\;\;\;\;\;\;\;\;\;\;\;\;\;\;\;\;\;\;\;\;\;\;\;\;\;\;\;\;\;\;-0.011^{*}Harmean-0.1251^{*}Skewness \end{array}$$

The memory usage level prediction formula of AB algorithm on mixed variable datasets is shown in formula (31).

(31) $$\begin{array}{l} \log_{10}{\left(Memory\right)}_{AB}=6.535+0.000002^{*}N+2.05^{*}R\_binary\\ \;\;\;\;\;\;\;\;\;\;\;\;\;\;\;\;\;\;\;\;\;\;\;\;\;\;\;\;\;\;\;\;\;\;\;\;\;\;\;\;\;\;\;\;\;\;\;\;\;\;\;+0.5903^{*}R\_largest-1.148^{*}R\_discrete\\ \;\;\;\;\;\;\;\;\;\;\;\;\;\;\;\;\;\;\;\;\;\;\;\;\;\;\;\;\;\;\;\;\;\;\;\;\;\;\;\;\;\;\;\;\;\;\;\;\;\;\;\;+1.454^{*}E\_C \end{array}$$

The memory usage level prediction formula of kNN algorithm on mixed variable datasets is shown in formula (32).

(32) $$\begin{array}{l} \log_{10}{\left(Memory\right)}_{kNN}=5.206+0.000003*N-1.84*R\_least\\ \;\;\;\;\;\;\;\;\;\;\;\;\;\;\;\;\;\;\;\;\;\;\;\;\;\;\;\;\;\;\;\;\;\;\;\;\;\;\;\;\;\;\;\;\;\;\;\;\;\;\;\;\;+1.174*R\_largest \end{array}$$

The memory usage level prediction formula of NB algorithm on mixed variable datasets is shown in formula (33).

(33) $$\log_{10}{\left(Memory\right)}_{NB}=4.559+0.0018^{*}P-0.0000005^{*}N$$

The memory usage level prediction formula of RF algorithm on mixed variable datasets is shown in formula (34).

(34) $$\begin{array}{l} \log_{10}{\left(Memory\right)}_{RF}=6.851+0.000002^{*}N-1.088^{*}R\_least\\ \;\;\;\;\;\;\;\;\;\;\;\;\;\;\;\;\;\;\;\;\;\;\;\;\;\;\;\;\;\;\;\;\;\;\;\;\;\;\;\;\;\;\;\;\;\;\;\;\;\;\;-0.008^{*}Harmean \end{array}$$

The memory usage level prediction formula of BP algorithm on mixed variable datasets is shown in formula (35).

(35) $$\begin{array}{l} \log_{10}{\left(Memory\right)}_{BP}=2.306+0.000003*N+3.272*R\_largest\\ \;\;\;\;\;\;\;\;\;\;\;\;\;\;\;\;\;\;\;\;\;\;\;\;\;\;\;\;\;\;\;\;\;\;\;\;\;\;\;\;\;\;\;\;\;\;\;\;\;\;+0.7722*MAr+2.417*E\_C\\ \;\;\;\;\;\;\;\;\;\;\;\;\;\;\;\;\;\;\;\;\;\;\;\;\;\;\;\;\;\;\;\;\;\;\;\;\;\;\;\;\;\;\;\;\;\;\;\;\;\;-6.748*Harmean \end{array}$$

#### 3.4.2 Discrete variable datasets

The memory usage level prediction formula of LR algorithm on discrete variable datasets is shown in formula (36).

(36) $$\log_{10}{\left(Memory\right)}_{LR}=4.872+0.00004^{*}N+0.0346^{*}N\_class$$

The memory usage level prediction formula of C4.5 algorithm on discrete variable datasets is shown in formula (37).

(37) $$\log_{10}{\left(Memory\right)}_{C4.5}=4.5535+0.0027^{*}P$$

The memory usage level prediction formula of SVM algorithm on discrete variable datasets is shown in formula (38).

(38) $$\begin{array}{l} \log_{10}{\left(Memory\right)}_{SVM}=5.668+0.00004*N+0.0246*N\_class\\ \;\;\;\;\;\;\;\;\;\;\;\;\;\;\;\;\;\;\;\;\;\;\;\;\;\;\;\;\;\;\;\;\;\;\;\;\;\;\;\;\;\;\;\;\;\;\;\;\;\;\;\;\;\;\;-1.206*R\_largest \end{array}$$

The memory usage level prediction formula of AB algorithm on discrete variable datasets is shown in formula (39).

(39) $$\begin{array}{l} \log_{10}{\left(Memory\right)}_{AB}=7.115+0.00004^{*}N+0.0021^{*}P\\ \;\;\;\;\;\;\;\;\;\;\;\;\;\;\;\;\;\;\;\;\;\;\;\;\;\;\;\;\;\;\;\;\;\;\;\;\;\;\;\;\;\;\;\;\;\;\;\;\;+0.0041^{*}ENV \end{array}$$

The memory usage level prediction formula of kNN algorithm on discrete variable datasets is shown in formula (40).

(40) $$\begin{array}{l} \log_{10}{\left(Memory\right)}_{kNN}=4.891+0.00004^{*}N+0.0152^{*}N\_class\\ \;\;\;\;\;\;\;\;\;\;\;\;\;\;\;\;\;\;\;\;\;\;\;\;\;\;\;\;\;\;\;\;\;\;\;\;\;\;\;\;\;\;\;\;\;\;\;\;\;\;\;\;\;+0.0019^{*}P \end{array}$$

The memory usage level prediction formula of NB algorithm on discrete variable datasets is shown in formula (41).

(41) $$\begin{array}{l} \log_{10}{\left(Memory\right)}_{NB}=5.1462+0.0018^{*}P-1.6206^{*}ME\_V\\ \;\;\;\;\;\;\;\;\;\;\;\;\;\;\;\;\;\;\;\;\;\;\;\;\;\;\;\;\;\;\;\;\;\;\;\;\;\;\;\;\;\;\;\;\;\;\;\;\;\;\;\;-0.3956^{*}R\_binary+0.0119^{*}N\_class\\ \;\;\;\;\;\;\;\;\;\;\;\;\;\;\;\;\;\;\;\;\;\;\;\;\;\;\;\;\;\;\;\;\;\;\;\;\;\;\;\;\;\;\;\;\;\;\;\;\;\;\;+0.0026^{*}NSR \end{array}$$

The memory usage level prediction formula of RF algorithm on discrete variable datasets is shown in formula (42).

(42) $$\log_{10}{\left(Memory\right)}_{RF}=5.872+0.00004^{*}N+0.6054^{*}E\_C$$

The memory usage level prediction formula of BP algorithm on discrete variable datasets is shown in formula (43).

(43) $$\log_{10}{\left(Memory\right)}_{BP}=4.715+0.00004^{*}N+0.0407^{*}N\_class$$

#### 3.4.3 Continuous variable datasets

The memory usage level prediction formula of LR algorithm on continuous variable datasets is shown in formula (44).

(44) $$\begin{array}{l} \log_{10}{\left(Memory\right)}_{LR}=5.913+0.000008^{*}N-1.76^{*}R\_least\\ \;\;\;\;\;\;\;\;\;\;\;\;\;\;\;\;\;\;\;\;\;\;\;\;\;\;\;\;\;\;\;\;\;\;\;\;\;\;\;\;\;\;\;\;\;\;\;\;\;\;+0.0000006^{*}Geomean-2.448^{*}MAr \end{array}$$

The memory usage level prediction formula of C4.5 algorithm on continuous variable datasets is shown in formula (45).

(45) $$\begin{array}{l} \log_{10}{\left(Memory\right)}_{C4.5}=4.626+0.000004^{*}N\\ \;\;\;\;\;\;\;\;\;\;\;\;\;\;\;\;\;\;\;\;\;\;\;\;\;\;\;\;\;\;\;\;\;\;\;\;\;\;\;\;\;\;\;\;\;\;\;\;\;\;\;\;\;+0.0000004^{*}Geomean\\ \;\;\;\;\;\;\;\;\;\;\;\;\;\;\;\;\;\;\;\;\;\;\;\;\;\;\;\;\;\;\;\;\;\;\;\;\;\;\;\;\;\;\;\;\;\;\;\;\;\;\;\;\;+0.0036^{*}P-0.0754^{*}Skewness \end{array}$$

The memory usage level prediction formula of SVM algorithm on continuous variable datasets is shown in formula (46).

(46) $$\log_{10}{\left(Memory\right)}_{SVM}=5.996+0.000008^{*}N-1.687^{*}R\_least$$

The memory usage level prediction formula of AB algorithm on continuous variable datasets is shown in formula (47).

(47) $$\begin{array}{l} \log_{10}{\left(Memory\right)}_{AB}=7.588+0.000006^{*}N\\ \;\;\;\;\;\;\;\;\;\;\;\;\;\;\;\;\;\;\;\;\;\;\;\;\;\;\;\;\;\;\;\;\;\;\;\;\;\;\;\;\;\;\;\;\;\;\;\;\;\;+0.0000005^{*}Geomean\\ \;\;\;\;\;\;\;\;\;\;\;\;\;\;\;\;\;\;\;\;\;\;\;\;\;\;\;\;\;\;\;\;\;\;\;\;\;\;\;\;\;\;\;\;\;\;\;\;\;\;\;\;-1.6^{*}MAr-0.7407^{*}R\_least \end{array}$$

The memory usage level prediction formula of kNN algorithm on continuous variable datasets is shown in formula (48).

(48) $$\begin{array}{l} \log_{10}{\left(Memory\right)}_{kNN}=5.916+0.000008^{*}N\\ \;\;\;\;\;\;\;\;\;\;\;\;\;\;\;\;\;\;\;\;\;\;\;\;\;\;\;\;\;\;\;\;\;\;\;\;\;\;\;\;\;\;\;\;\;\;\;\;\;\;\;\;\;+0.0000007^{*}Geomean\\ \;\;\;\;\;\;\;\;\;\;\;\;\;\;\;\;\;\;\;\;\;\;\;\;\;\;\;\;\;\;\;\;\;\;\;\;\;\;\;\;\;\;\;\;\;\;\;\;\;\;\;\;\;\;-1.515^{*}R\_least-2.27^{*}MAr \end{array}$$

The memory usage level prediction formula of NB algorithm on continuous variable datasets is shown in formula (49).

(49) $$\begin{array}{l} \log_{10}{\left(Memory\right)}_{NB}=4.0255+0.0053^{*}P+0.0088^{*}N\_class\\ \;\;\;\;\;\;\;\;\;\;\;\;\;\;\;\;\;\;\;\;\;\;\;\;\;\;\;\;\;\;\;\;\;\;\;\;\;\;\;\;\;\;\;\;\;\;\;\;\;\;\;+0.2934^{*}E\_C \end{array}$$

The memory usage level prediction formula of RF algorithm on continuous variable datasets is shown in formula (50).

(50) $$\begin{array}{l} \log_{10}{\left(Memory\right)}_{RF}=6.806+0.000005^{*}N\\ \;\;\;\;\;\;\;\;\;\;\;\;\;\;\;\;\;\;\;\;\;\;\;\;\;\;\;\;\;\;\;\;\;\;\;\;\;\;\;\;\;\;\;\;\;\;\;\;\;\;+0.0000008^{*}Geomean\\ \;\;\;\;\;\;\;\;\;\;\;\;\;\;\;\;\;\;\;\;\;\;\;\;\;\;\;\;\;\;\;\;\;\;\;\;\;\;\;\;\;\;\;\;\;\;\;\;\;\;\;-1.186^{*}R\_least-1.637^{*}MAr \end{array}$$

The memory usage level prediction formula of BP algorithm on continuous variable datasets is shown in formula (51).

(51) $$\begin{array}{l} \log_{10}{\left(Memory\right)}_{BP}=5.83+0.000008^{*}N-1.986^{*}R\_least\\ \;\;\;\;\;\;\;\;\;\;\;\;\;\;\;\;\;\;\;\;\;\;\;\;\;\;\;\;\;\;\;\;\;\;\;\;\;\;\;\;\;\;\;\;\;\;\;\;\;\;+0.0000006^{*}Geomean-2.158^{*}MAr \end{array}$$

## 4 Discussion

**Figure 3:**
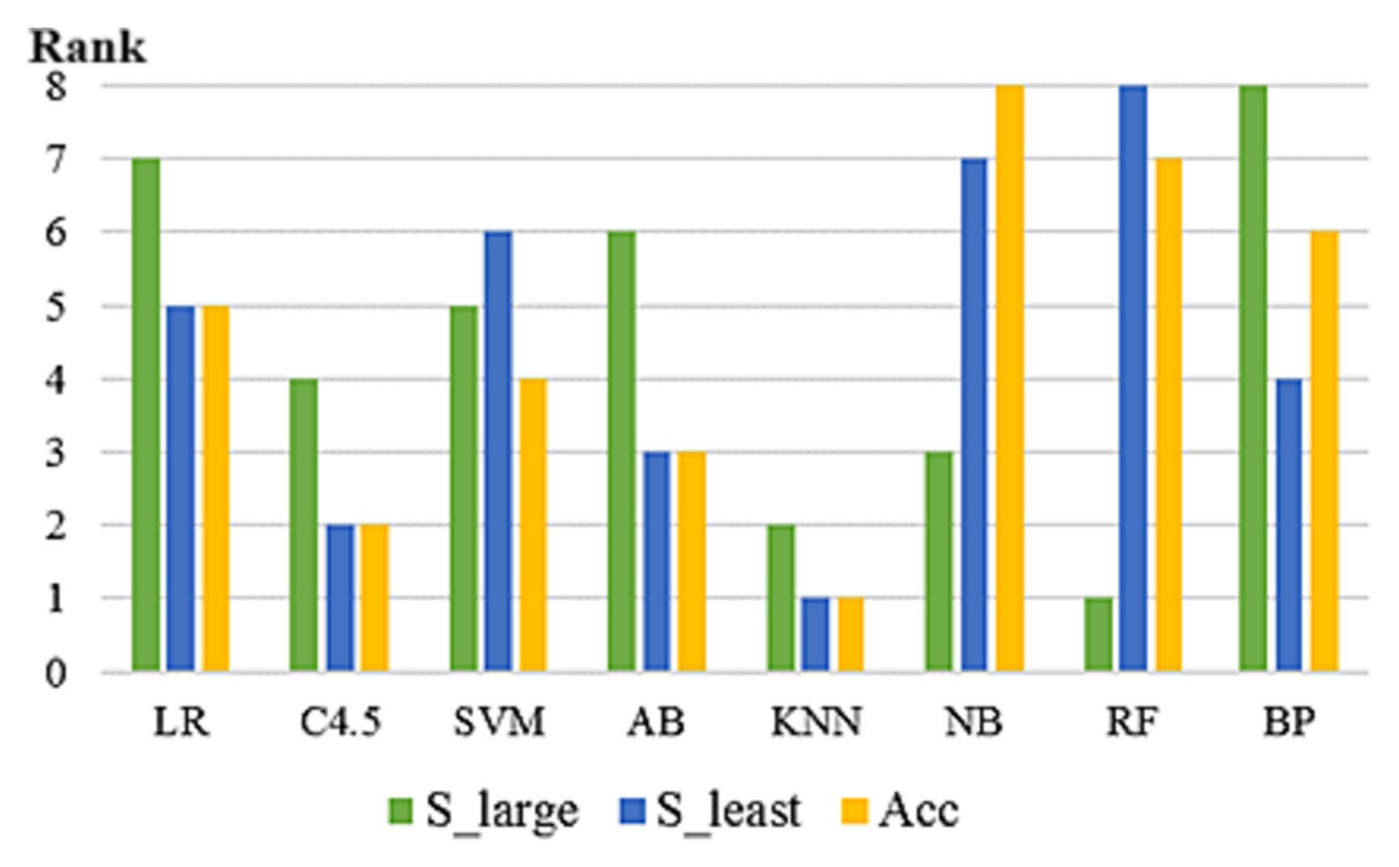
Prediction accuracy ranking of 8 algorithms under different evaluation indexes (on the dataset “Abalone”).

In 293 UCI data subsets included in the study, the rankings of the eight algorithms varied according to three prediction accuracy evaluation indicators. As can be seen from Figure 3, on some datasets, the rankings of the eight algorithms vary among different evaluation indicators. No algorithm can maintain the optimal position under any evaluation index framework, which proves the scientific nature of NFL theorem and the necessity of this paper. In addition, on some datasets, several algorithms reach the optimal level at the same time.

In 2000, Lim et al. found that among decision tree algorithms, C4.5, IND-CART and QUEST had a better balance between accuracy and speed, but C4.5 tended to generate trees twice or larger than the latter two. Among statistical algorithms, Logistic regression algorithm has a more prominent performance (Smith, 2008).

Based on the results of exploratory analysis of the above three types of datasets and the results of evaluation and comparison of stepwise regression and decision tree modeling results, the following knowledge of algorithm applicability based on prediction accuracy can be obtained.

### 4.1 Mixed variable datasets

The performance of AB, NB and BP algorithms on datasets from different fields will be different. AB algorithm is suitable for medical datasets with discrete variable ratio less than 77.78%, and NB algorithm is suitable for datasets with noise to signal ratio greater than −38.7407.

### 4.2 Discrete variable datasets

The performance of NB algorithm on datasets in biomedical field is obviously better than that on datasets in general field. Due to the small number of discrete variable datasets in the UCI public dataset, other algorithms did not show a statistically significant performance gap on the discrete variable datasets included in the study.

### 4.3 Continuous variable datasets

**Figure 4:**
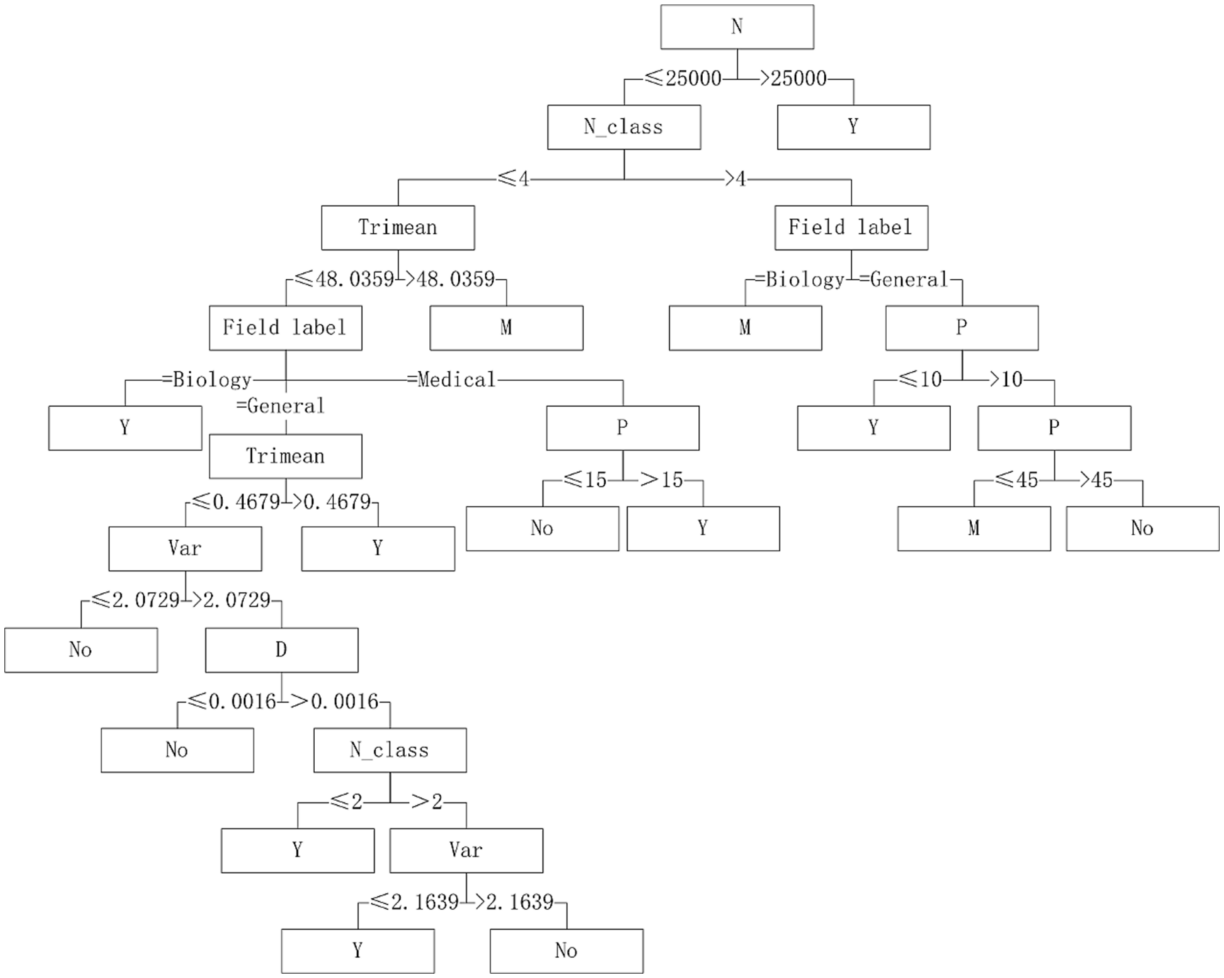
Decision tree model of C4.5 algorithm applicability – continuous variable data sets.

**Figure 5:**
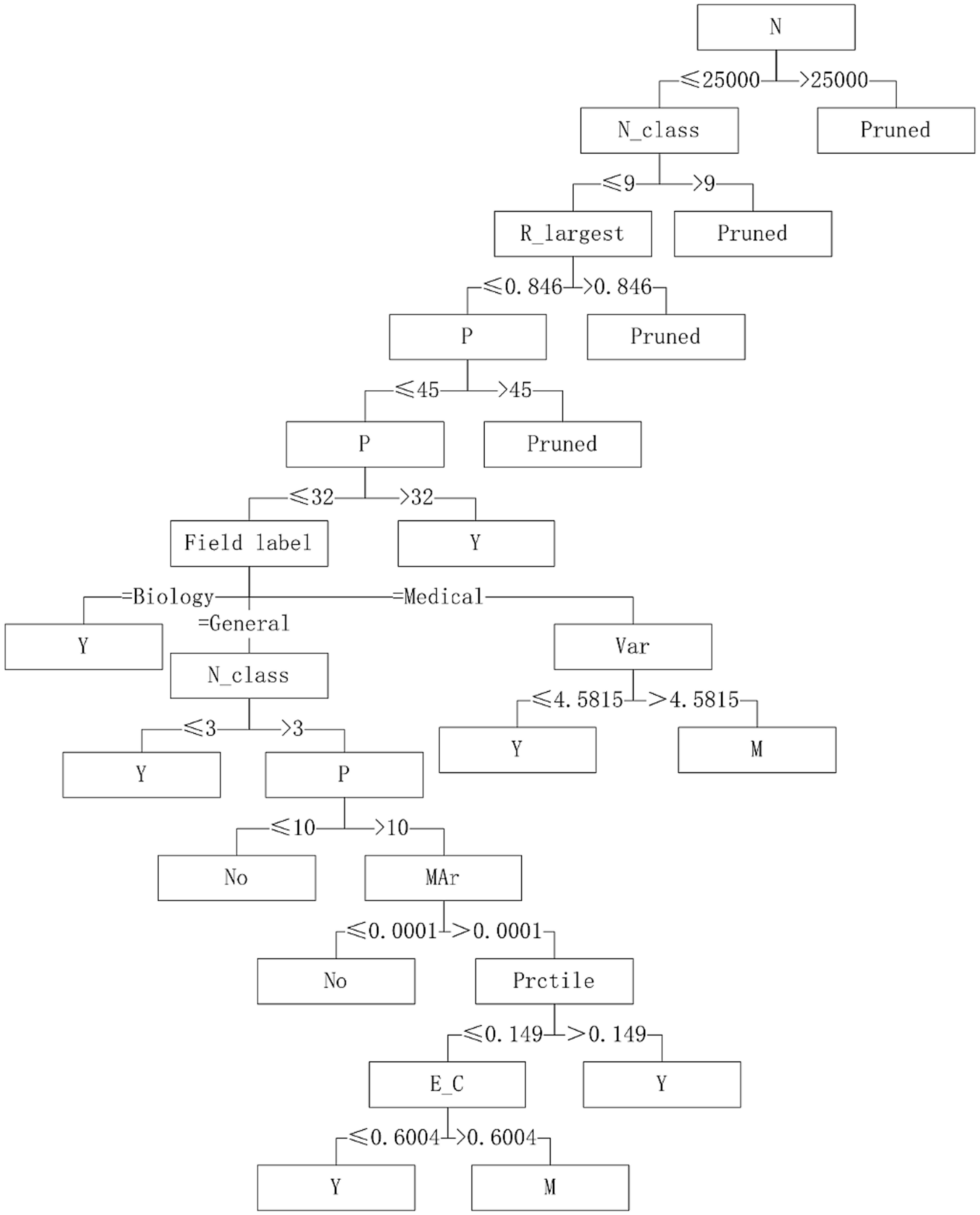
Decision tree model of SVM algorithm applicability – continuous variable data sets.

**Figure 6:**
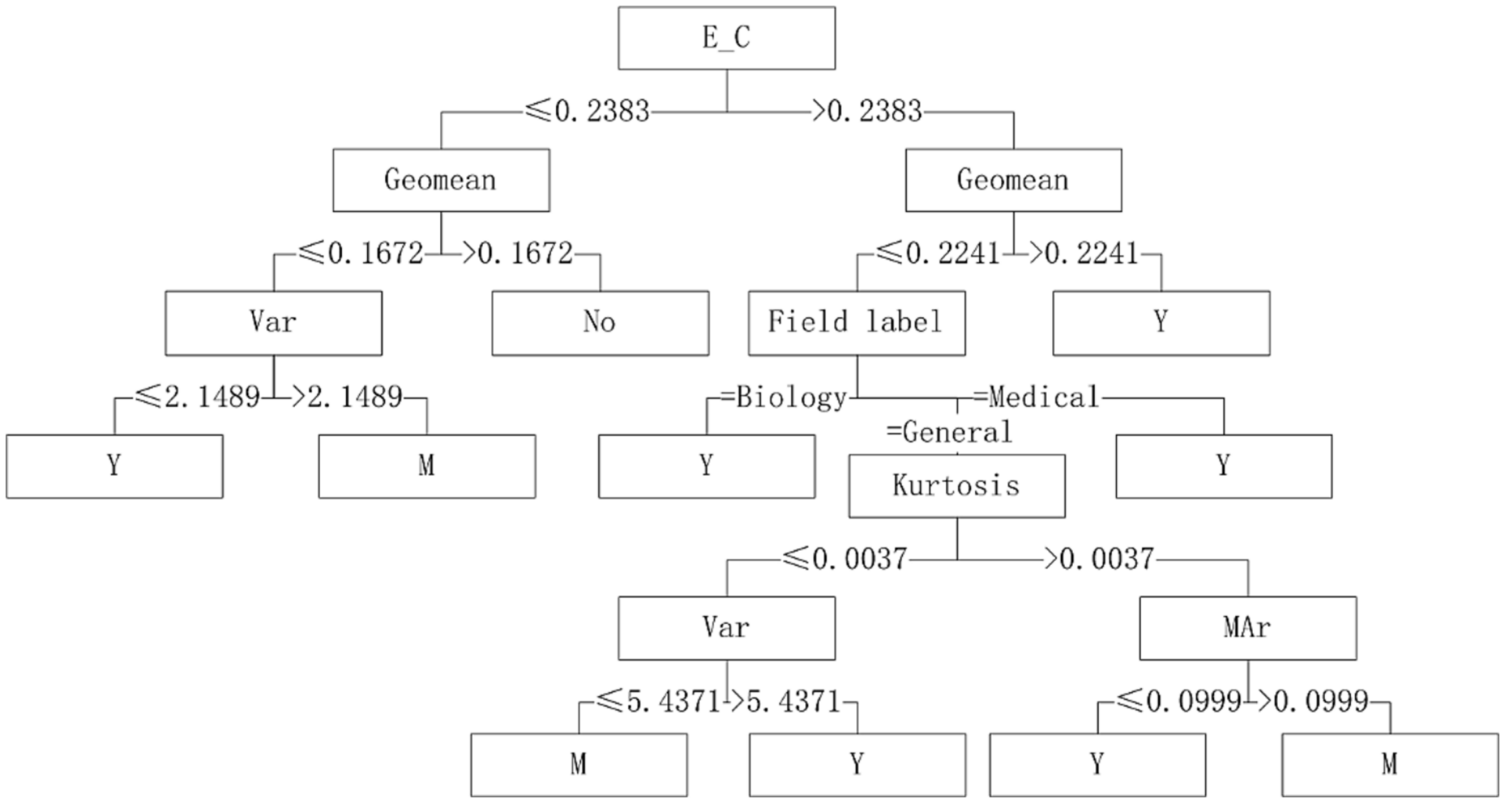
Decision tree model of RF algorithm applicability – continuous variable data sets.

Five algorithms, C4.5, SVM, NB, RF and BP, have different performance on datasets from different fields. The C4.5 algorithm is recommended for medical datasets with more than 15 variables. For medical datasets whose mean variance of variables is less than or equal to 4.5815, SVM algorithm can be considered. The RF algorithm is considered for medical datasets with the information entropy of class variables greater than 0.2383 and the geometric mean less than or equal to 0.2241. The corresponding decision tree model is shown in Figures 4–6.

From formulas (4)–(27), it can be found that sample size N is an important factor affecting the modeling running time of each base algorithm on the three types of datasets. In addition, the running time of each base algorithm on the mixed variable datasets is also related to R_least and Harmean. The running time of each base algorithm on continuous variable datasets is mainly related to R_least and Geomean.

From formulas (28)–(51), it can be found that sample size N is an important factor affecting the modeling memory of each base algorithm on the three types of datasets. In addition, the memory usage of each base algorithm on the mixed variable datasets is also related to R_least and R_largest. The memory usage of each base algorithm on discrete variable datasets is mainly related to N_class. The memory usage of each base algorithm on continuous variable datasets is related to R_least and Geomean.

## 5 Conclusion

The validity and feasibility of the algorithm applicability knowledge base constructed in this paper have been verified theoretically, thus realizing the construction of the algorithm applicability knowledge base of the dataset oriented to classification task. Compared with other studies, this paper focuses the problem space of algorithm applicability in the medical field for the first time, and it is found that C4.5 algorithm has outstanding performance on most medical datasets, ranking in the forefront of prediction accuracy, comparable to the ensemble methods, and the order of magnitude modeling running time and memory occupation is relatively smaller.

As for the applicability of data mining algorithms, although this paper has carried out a relatively in-depth analysis by introducing algorithm selection concept, algorithm recommendation and meta-learning theory, expected to obtain rule knowledge with guiding value for medical data mining practice. However, due to the limitations of theory and practice, this paper still has some shortcomings and needs further research. All kinds of specific problems in the biomedical field can be abstractions into classification, numerical prediction, clustering, association rules and time series analysis in data mining, and 70% of problems in real life can be transformed into classification problems. In this paper, the applicability of the algorithm is studied only in the field of classification tasks, and subsequent studies can expand the breadth of mining tasks, such as continuing to study the applicability of the algorithm in the field of numerical prediction tasks, the applicability of various deep neural networks in medical image analysis, the influence of data preprocessing methods on modeling results, etc.

## Data availability statement

The original contributions presented in the study are included in the article/supplementary material, further inquiries can be directed to the corresponding author.

## Author contributions

YZ: Formal analysis, Funding acquisition, Methodology, Writing – original draft. QL: Conceptualization, Writing – review & editing. YX: Conceptualization, Writing – review & editing.
